# Supplementary material for: Phase vortex lattices in neutron interferometry
Source: Commun Phys. 2023 Aug 9;6(1):209. doi: 10.1038/s42005-023-01318-6 (PMC11041680; doi:10.1038/s42005-023-01318-6)
Supplement: Supplementary file 1 — Supplementary Information [file 42005_2023_1318_MOESM1_ESM.pdf]

Supplementary Information: Phase Vortex Lattices in Neutron Interferometry

Niels Geerits[1], Hartmut Lemmel[1,2], Anna-Sophie Berger[1], and Stephan Sponar[1]
[1]*Atominstitut, Technische Universität Wien, Stadionallee 2, 1020 Vienna, Austria*
[2]*Institut Laue-Langevin, 71 Avenue des Martyrs, CS 20156, 38042 Grenoble Cedex 9, France*

SUPPLEMENTARY NOTE 1: DETAILED CALCULATIONS

In this supplement the step by step calculations of $< L_z >$ (for isotropic and anisotropic momentum distributions) and $< L_z^2 >$ are shown. Starting with the calculation of $< L_z >$ in cylindrical coordinates

$$< L_z >= -\mathrm{i}\frac{\int \mathrm{d}\mathbf{r}\psi_t^*(\mathbf{r})\frac{\partial}{\partial\phi}\psi(\mathbf{r})}{\int \mathrm{d}\mathbf{r}|\psi_t(\mathbf{r})|^2}$$

with

$$\int \mathrm{d}\mathbf{r}|\psi_t(\mathbf{r})|^2 = 1+\cos(\Delta\alpha)e^{-\frac{\sigma^2 k_\perp^2}{4}} = N$$

and

$$-\mathrm{i}\frac{\partial}{\partial\phi}\psi_t(\mathbf{r}) = \frac{1}{\sqrt{2}}\psi_0[k_\perp\rho\cos(\phi)e^{\mathrm{i}k_\perp\rho\sin(\phi)} - e^{\mathrm{i}\Delta\alpha}k_\perp\rho\sin(\phi)e^{\mathrm{i}k_\perp\rho\cos(\phi)}]$$

hence it follows

$$< L_z >= \frac{1}{2N}\int \mathrm{d}\mathbf{r}|\psi_0|^2[k_\perp\rho\cos(\phi) - k_\perp\rho\sin(\phi) + k_\perp\rho\cos(\phi)e^{-\mathrm{i}\Delta\alpha}e^{\mathrm{i}k_\perp\rho(\sin(\phi)-\cos(\phi))} - k_\perp\rho\sin(\phi)e^{\mathrm{i}\Delta\alpha}e^{\mathrm{i}k_\perp\rho(\cos(\phi)-\sin(\phi))}]$$

which, using $\int_0^{2\pi}\mathrm{d}\phi\cos(\phi) = \int_0^{2\pi}\mathrm{d}\phi\sin(\phi) = 0$, simplifies to

$$< L_z >= \frac{1}{2N}\int \mathrm{d}\mathbf{r}k_\perp\rho|\psi_0|^2[\cos(\phi)e^{-\mathrm{i}\Delta\alpha}e^{\mathrm{i}k_\perp\rho(\sin(\phi)-\cos(\phi))} - \sin(\phi)e^{\mathrm{i}\Delta\alpha}e^{\mathrm{i}k_\perp\rho(\cos(\phi)-\sin(\phi))}]$$

$$< L_z >= \frac{1}{2N}\int d\mathbf{r}k_\perp\rho|\psi_0|^2[\cos(\phi)e^{-i\Delta\alpha}e^{\mathrm{i}\sqrt{2}k_\perp\rho\sin(\phi-\pi/4)} - \sin(\phi)e^{\mathrm{i}\Delta\alpha}e^{-i\sqrt{2}k_\perp\rho sin(\phi-\pi/4)}]$$

Then we apply the Jacobi-Anger expansion, $e^{\mathrm{i}z\sin(\phi)} = \sum_\ell J_\ell(z)e^{\mathrm{i}\ell\phi}$, and use that $\int_0^{2\pi}\mathrm{d}\phi e^{\mathrm{i}\ell\phi} = 0$ for $\ell \neq 0$ This allows us to easily solve the azimuthal integral.

$$< L_z >= \frac{\pi}{2N}\int \mathrm{d}\rho k_\perp\rho^2|\psi_0|^2[J_{-1}(\sqrt{2}k_\perp\rho)e^{-\mathrm{i}\Delta\alpha}e^{\mathrm{i}\frac{\pi}{4}} + J_1(\sqrt{2}k_\perp\rho)e^{-\mathrm{i}\Delta\alpha}e^{-\mathrm{i}\frac{\pi}{4}} -$$

$$\mathrm{i}J_{-1}(\sqrt{2}k_\perp\rho)e^{\mathrm{i}\Delta\alpha}e^{\mathrm{i}\frac{\pi}{4}} + \mathrm{i}J_1(\sqrt{2}k_\perp\rho)e^{\mathrm{i}\Delta\alpha}e^{-\mathrm{i}\frac{\pi}{4}}]$$

Next we use the anti-symmetry of the Bessel function of first order $J_{-1}(z) = -J_1(z)$ and begin grouping the exponential/trigonometric terms.

$$< L_z >= -\mathrm{i}\frac{\pi}{N}\int \mathrm{d}\rho k_\perp\rho^2|\psi_0|^2 J_1(\sqrt{2}k_\perp\rho)[e^{-\mathrm{i}\Delta\alpha}\sin(\frac{\pi}{4}) - e^{\mathrm{i}\Delta\alpha}\cos(\frac{\pi}{4})]$$

$$< L_z >= 2\pi\frac{\sin\Delta\alpha}{\sqrt{2}N}\int \mathrm{d}\rho k_\perp\rho^2|\psi_0|^2 J_1(\sqrt{2}k_\perp\rho)$$

Which can be rewritten into the form of a standard Hankel transform of first order with known result. This brings us to the equation (8) seen in the main text

$$< L_z >= \sin(\Delta\alpha)\frac{k_\perp^2\sigma^2}{4N}e^{-\frac{\sigma^2 k_\perp^2}{4}}$$

Next we examine the generalized case where the momentum distribution is anisotropic (see equation (13) in the main text). This is best done in Cartesian coordinates:

$$L_z\psi_t = -\mathrm{i}(x\frac{\partial}{\partial y} - y\frac{\partial}{\partial x})\psi_t$$

$$= -\frac{\mathrm{i}}{\sqrt{\pi\sigma_x\sigma_y}}e^{-\frac{x^2}{\sigma_x^2}-\frac{y^2}{\sigma_y^2}}(2xy(e^{\mathrm{i}k_\perp y} + e^{\mathrm{i}\Delta\alpha}e^{\mathrm{i}k_\perp x})[\frac{1}{\sigma_x^2} - \frac{1}{\sigma_y^2}] + \mathrm{i}k_\perp x e^{\mathrm{i}k_\perp y} - \mathrm{i}k_\perp y e^{\mathrm{i}\Delta\alpha}e^{\mathrm{i}k_\perp x})$$

Hence it follows

$$< L_z >= \frac{k_\perp}{\pi\sigma_x\sigma_y N}\int \mathrm{d}x\mathrm{d}y e^{-2\frac{x^2}{\sigma_x^2}-2\frac{y^2}{\sigma_y^2}}[xe^{-\mathrm{i}k_\perp(x-y)-\mathrm{i}\Delta\alpha} - ye^{\mathrm{i}k_\perp(x-y)+\mathrm{i}\Delta\alpha}]$$

Where all odd terms have been dropped since their integral is zero. To proceed we use $\mathrm{i}\frac{\partial}{\partial k}e^{-\mathrm{i}k(a+b)} = (a+b)e^{-\mathrm{i}k(a+b)}$ to get

$$< L_z >= \frac{k_\perp}{\pi\sigma_x\sigma_y N}\int \mathrm{d}x\mathrm{d}y e^{-2\frac{x^2}{\sigma_x^2}-2\frac{y^2}{\sigma_y^2}}[\mathrm{i}\frac{\partial}{\partial k_\perp}e^{-\mathrm{i}k_\perp(x-y)-\mathrm{i}\Delta\alpha} - \mathrm{i}\frac{\partial}{\partial k}e^{\mathrm{i}k_\perp(x-y)+\mathrm{i}\Delta\alpha} + ye^{-\mathrm{i}k_\perp(x-y)-\mathrm{i}\Delta\alpha} - xe^{\mathrm{i}k_\perp(x-y)+\mathrm{i}\Delta\alpha}]$$

Note the final term is minus the complex conjugate of our previous expression for $< L_z >$ hence it follows

$$< L_z >= \frac{k_\perp}{\pi\sigma_x\sigma_y N}\int \mathrm{d}x\mathrm{d}y e^{-2\frac{x^2}{\sigma_x^2}-2\frac{y^2}{\sigma_y^2}}[\mathrm{i}\frac{\partial}{\partial k_\perp}e^{-\mathrm{i}k_\perp(x-y)-\mathrm{i}\Delta\alpha} - \mathrm{i}\frac{\partial}{\partial k}e^{\mathrm{i}k_\perp(x-y)+\mathrm{i}\Delta\alpha}] - < L_z >^*$$

and since expectation values must be real we can conclude

$$< L_z >= \frac{\mathrm{i}k_\perp}{2\pi\sigma_x\sigma_y N}\int \mathrm{d}x\mathrm{d}y e^{-2\frac{x^2}{\sigma_x^2}-2\frac{y^2}{\sigma_y^2}}\frac{\partial}{\partial k_\perp}[e^{-\mathrm{i}k_\perp(x-y)-\mathrm{i}\Delta\alpha} - e^{\mathrm{i}k_\perp(x-y)+\mathrm{i}\Delta\alpha}]$$

We may now swap integration and differentiation and realize that we are left with a standard Fourier transform

$$< L_z >= \frac{\mathrm{i}k_\perp}{2\pi\sigma_x\sigma_y N}\frac{\partial}{\partial k_\perp}\int \mathrm{d}x\mathrm{d}y e^{-2\frac{x^2}{\sigma_x^2}-2\frac{y^2}{\sigma_y^2}}[e^{-\mathrm{i}k_\perp(x-y)-\mathrm{i}\Delta\alpha} - e^{\mathrm{i}k_\perp(x-y)+\mathrm{i}\Delta\alpha}]$$

Conducting the transform and grouping the exponential/trigonometric terms leads to

$$< L_z >= \frac{k_\perp}{2N}\frac{\partial}{\partial k_\perp}e^{-\frac{(\sigma_x^2+\sigma_y^2)k^2}{8}}\sin(\Delta\alpha)$$

Finally carrying out the differentiation leads to the result shown in equation (14) of the main text

$$< L_z >= \sin(\Delta\alpha)\frac{k_\perp^2(\sigma_x^2+\sigma_y^2)}{8N}e^{-\frac{k_\perp^2(\sigma_x^2+\sigma_y^2)}{8}}$$

Finally we calculate the second moment of the OAM distribution, $< L_z^2 >$

$$< L_z^2 >= -\frac{\int d\mathbf{r}\psi_t^*(\mathbf{r})\frac{\partial^2}{\partial\phi^2}\psi_t(\mathbf{r})}{N}$$

$$\frac{\partial^2}{\partial\phi^2}\psi(\mathbf{r}) = -\frac{1}{\sqrt{2}}\psi_0[(k_\perp^2\rho^2\sin^2(\phi)+\mathrm{i}k_\perp\rho\cos(\phi))e^{\mathrm{i}\Delta\alpha}e^{\mathrm{i}k_\perp\rho\cos(\phi)} + (k_\perp^2\rho^2\cos^2(\phi)+\mathrm{i}k_\perp\rho\sin(\phi))e^{\mathrm{i}k_\perp\rho\sin(\phi)}]$$

Therefore

$$< L_z^2 >= \frac{1}{2N}\int \mathrm{d}\mathbf{r}|\psi_0|^2[(k_\perp^2\rho^2\sin^2(\phi)+\mathrm{i}k_\perp\rho\cos(\phi)) + (k_\perp^2\rho^2\cos^2(\phi)+\mathrm{i}k_\perp\rho\sin(\phi))+$$

$$(k_\perp^2\rho^2\sin^2(\phi)+\mathrm{i}k_\perp\rho\cos(\phi))e^{\mathrm{i}\Delta\alpha}e^{\mathrm{i}k_\perp\rho[\cos(\phi)-sin(\phi)]} + (k_\perp^2\rho^2\cos^2(\phi)+\mathrm{i}k_\perp\rho\sin(\phi))e^{-\mathrm{i}\Delta\alpha}e^{\mathrm{i}k_\perp\rho[\sin(\phi)-\cos(\phi)]}]$$

First we use $\cos(\phi)-\sin(\phi) = -\sqrt{2}\sin(\phi-\frac{\pi}{4})$

$$< L_z^2 >= \frac{1}{2N}\int \mathrm{d}\mathbf{r}|\psi_0|^2[k_\perp^2\rho^2\sin^2(\phi) + k_\perp^2\rho^2\cos^2(\phi)+$$

$$(k_\perp^2\rho^2\sin^2(\phi)+\mathrm{i}k_\perp\rho\cos(\phi))e^{\mathrm{i}\Delta\alpha}e^{-\mathrm{i}\sqrt{2}k_\perp\rho\sin(\phi-\frac{\pi}{4})} + (k_\perp^2\rho^2\cos^2(\phi)+\mathrm{i}k_\perp\rho\sin(\phi))e^{-\mathrm{i}\Delta\alpha}e^{\mathrm{i}\sqrt{2}k_\perp\rho\sin(\phi-\frac{\pi}{4})}]$$

We simplify the expression by using the identity $\cos^2(\phi)+\sin^2(\phi) = 1$

$$< L_z^2 >= \frac{1}{2N}\int \mathrm{d}\mathbf{r}|\psi_0|^2[k_\perp^2\rho^2 + (k_\perp^2\rho^2\sin^2(\phi)+\mathrm{i}k_\perp\rho\cos(\phi))e^{\mathrm{i}\Delta\alpha}e^{-\mathrm{i}\sqrt{2}k_\perp\rho\sin(\phi-\frac{\pi}{4})]}+$$

$$(k_\perp^2\rho^2\cos^2(\phi)+\mathrm{i}k_\perp\rho\sin(\phi))e^{-\mathrm{i}\Delta\alpha}e^{\mathrm{i}\sqrt{2}k_\perp\rho\sin(\phi-\frac{\pi}{4})}]$$

We solve the azimuthal integral by using use the Jacobi-Anger expansion again, $e^{\mathrm{i}z\sin(\phi)} = \sum_\ell J_\ell(z)e^{\mathrm{i}\ell\phi}$, and again use that $\int_0^{2\pi}\mathrm{d}\phi e^{\mathrm{i}\ell\phi} = 0$ for $\ell \neq 0$. Note that the latter identity paired with the trigonometric terms in the previous line filter out all but the $\ell = 0$ and $\ell = \pm 1$ terms of the Jacobi-Anger expansion.

$$< L_z^2 >= \frac{1}{2N}\int \mathrm{d}\rho\rho|\psi_0|^2[2\pi k_\perp^2\rho^2 + (\pi k_\perp^2\rho^2 J_0(\sqrt{2}k_\perp\rho) - \sqrt{2}\pi k_\perp\rho J_1(\sqrt{2}k_\perp\rho))e^{\mathrm{i}\Delta\alpha}+$$

$$(\pi k_\perp^2\rho^2 J_0(\sqrt{2}k_\perp\rho) - \sqrt{2}\pi k_\perp\rho J_1(\sqrt{2}k_\perp\rho))e^{-\mathrm{i}\Delta\alpha}]$$

Here we have once again used the asymmetry of the first order Bessel function. Next we group together the trigonometric terms

$$< L_z^2 >= \frac{1}{2N}\int \mathrm{d}\rho\rho|\psi_0|^2[2\pi k_\perp^2\rho^2 + \cos(\Delta\alpha)(2\pi k_\perp^2\rho^2 J_0(\sqrt{2}k_\perp\rho) - \sqrt{8}\pi k_\perp\rho J_1(\sqrt{2}k_\perp\rho)]$$

Now we attempt to solve the radial integrals

$$< L_z^2 >= \frac{1}{\pi\sigma^2 N}\int \mathrm{d}\rho\rho e^{-2\frac{\rho^2}{\sigma^2}}[2\pi k_\perp^2\rho^2 + \cos(\Delta\alpha)(2\pi k_\perp^2\rho^2 J_0(\sqrt{2}k_\perp\rho) - \sqrt{8}\pi k_\perp\rho J_1(\sqrt{2}k_\perp\rho)]$$

The first integral seen above:

$$\int_0^\infty \mathrm{d}\rho 2\pi k_\perp^2\rho^3 e^{-2\frac{\rho^2}{\sigma^2}}$$

can be solved using integration by parts and substitution ($u = \rho^2$ and $\mathrm{d}u = \rho\mathrm{d}\rho$)

$$\int_0^\infty \mathrm{d}u\pi k_\perp^2 u e^{-2\frac{u}{\sigma^2}} = [-\pi k_\perp^2\frac{u\sigma^2}{2}e^{-\frac{-2u}{\sigma^2}}]_0^\infty + \int_0^\infty \mathrm{d}u\pi k_\perp^2\frac{\sigma^2}{2}e^{-2\frac{u}{\sigma^2}} = \frac{\pi k_\perp^2\sigma^4}{4}$$

The next radial integral in $< L_z^2 >$ is a Hankel transform with a known result:

$$2\pi\cos(\Delta\alpha)\int_0^\infty \mathrm{d}\rho k_\perp^2 \rho^3 e^{-2\frac{\rho^2}{\sigma^2}} J_0(\sqrt{2}k_\perp\rho) = \frac{\pi k_\perp^2\sigma^4}{4}\cos(\Delta\alpha)e^{-\frac{\sigma^2 k_\perp^2}{4}}(1-\frac{k_\perp^2\sigma^2}{4})$$

The final integral is the same Hankel transform as for the first moment

$$-\sqrt{8}\pi\cos(\Delta\alpha)\int d\rho e^{-2\frac{\rho^2}{\sigma^2}} k_\perp\rho^2 J_1(\sqrt{2}k_\perp\rho) = -\cos(\Delta\alpha)\frac{\pi k_\perp^2\sigma^4}{4}e^{-\frac{k_\perp^2\sigma^2}{4}}$$

Hence we find

$$< L_z^2 >= \frac{k_\perp^2\sigma^2}{4N} + \cos(\alpha)\frac{k_\perp^2\sigma^2}{4N}e^{-\frac{\sigma^2 k_\perp^2}{4}}(1-\frac{k_\perp^2\sigma^2}{4}) - \cos(\alpha)\frac{k_\perp^2\sigma^2}{4N}e^{-\frac{k_\perp^2\sigma^2}{4}}$$

$$< L_z^2 >= \frac{k_\perp^2\sigma^2}{4N} - \cos(\alpha)\frac{k_\perp^4\sigma^4}{16N}e^{-\frac{\sigma^2 k_\perp^2}{4}}$$
